# Using deep learning for pruning region detection and plant organ segmentation in dormant spur-pruned grapevines

**DOI:** 10.1007/s11119-023-10006-y

**Published:** 2023-03-22

**Authors:** P. Guadagna, M. Fernandes, F. Chen, A. Santamaria, T. Teng, T. Frioni, D. G. Caldwell, S. Poni, C. Semini, M. Gatti

**Affiliations:** 1 Department of Sustainable Crop Production (DI.PRO.VE.S.), Università Cattolica del Sacro Cuore, Via Emilia Parmense 84, 29122 Piacenza, Italy; 2 Istituto Italiano di Tecnologia, Via S. Quirico 19D, 16163 Genoa, Italy

**Keywords:** Computer vision, Object detection, Robotics, Viticulture, Winter pruning

## Abstract

Even though mechanization has dramatically decreased labor requirements, vineyard management costs are still affected by selective operations such as winter pruning. Robotic solutions are becoming more common in agriculture, however, few studies have focused on grapevines. This work aims at fine-tuning and testing two different deep neural networks for: (i) detecting pruning regions (PRs), and (ii) performing organ segmentation of spur-pruned dormant grapevines. The Faster R-CNN network was fine-tuned using 1215 RGB images collected in different vineyards and annotated through bounding boxes. The network was tested on 232 RGB images, PRs were categorized by wood type (W), orientation (Or) and visibility (V), and performance metrics were calculated. PR detection was dramatically affected by visibility. Highest detection was associated with visible intermediate complex spurs in Merlot (0.97), while most represented coplanar simple spurs allowed a 74% detection rate. The Mask R-CNN network was trained for grapevine organs (GOs) segmentation by using 119 RGB images annotated by distinguishing 5 classes (cordon, arm, spur, cane and node). The network was tested on 60 RGB images of light pruned (LP), shoot-thinned (ST) and unthinned control (C) grapevines. Nodes were the best segmented GOs (0.88) and general recall was higher for ST (0.85) compared to C (0.80) confirming the role of canopy management in improving performances of hi-tech solutions based on artificial intelligence. The two fine-tuned and tested networks are part of a larger control framework that is under development for autonomous winter pruning of grapevines.

**Supplementary Information:**

The online version contains supplementary material available at 10.1007/s11119-023-10006-y.

## Introduction

Viticulture covers more than 7.3 million hectares around the world. Grapevines represent a major Mediterranean permanent crop especially for countries such as Spain, France and Italy, accounting for about 50% of total acreage; in parallel, the wine industry is widely growing in the so-called New World wine-producing countries including China, USA, New Zealand and Australia (OIV, 2021). Due to unfavorable topographic conditions (i.e. steep slopes), in several wine districts mechanization is troublesome and vineyard management mostly relies on manual operations such as winter and summer pruning, bunch and shoot thinning, and harvesting (Poni et al., 2018). Regardless of the site characteristics, labor is the major cost in vineyard management with harvest and winter pruning representing the most expensive and time-consuming operations (Intrieri & Poni, 1995).

Moreover, job opportunities in other sectors of the economy are becoming more attractive for a large part of the workforce previously involved in agriculture, and several world regions are experiencing an overall shortage of skilled workers (Charlton & Taylor, 2016; Eurostat, 2021). A condition that has recently been exacerbated by international restrictions to mobility related to the Covid-19 pandemic, with increased competition for the limited availability of skilled operators, and vineyard managers concerned to secure seasonal workers joining their vineyard crews (Rivera-Ferre et al., 2021; Squire, 2020). Finally, the use of shears in repetitive gestures increases the risk of injuries for the operator (Fathallah, 2010).

To address the overall shortage of vineyard labor and to increase competitiveness of the wine industry, mechanical pruning has been investigated by several researchers (Clingeleffer, 2013; Dokoozlian, 2013; Intrieri, 2013; Poni et al., 2016; Shaulis et al., 1967). In particular, spur pruning can be partially mechanized by using cutting bars or rotating disks to remove the previous season’s growth as well as to cut dormant shoots into small fragments that fall on the ground, with a significant decrease in labour requirements for performing rough cuts and cane stripping. However, VSP-trained spurred cordons is not the only approach suitable for mechanical pruning, and other training systems have been specifically developed for a more intensive or full mechanization: such as single-wire cordon, Geneva Double Courtain and Lyra (Intrieri & Poni, 1995; Intrieri et al., 2011). As an example, mechanical pruning of single-wire cordon trained Barbera grapevines was effective in reducing the labour demand from 60 h/ha to 25 and 17 h/ha depending on different intensities of manual follow-up (Gatti et al., 2011), whilst minimal pruning was completed in less than 20 h/ha in Australian vineyards (Clingeleffer, 2013). However, the most advanced spur pruning technology is still represented by non–selective mechanical operations requiring manual follow-up (Poni et al., 2016), and the use of heavy combustion-engine powered tractors in vineyards contributes to soil compaction and increases the overall carbon footprint (Longbottom & Petrie, 2015; Pessina et al., 2021).

Given the increasing competitiveness of the wine business on a global scale, new efficient solutions for vineyard management are therefore required to create integrated sustainability (Christ & Burritt, 2013; Rugani et al., 2013; Tardaguila et al., 2021). Over the past few decades, advances in technology have contributed to improving final quality and efficiency in several agricultural systems. Precision viticulture protocols have been developed since the ‘90 s (Bramley, 2010) and variable rate technologies can now assist cultural practices such as irrigation (Sanchez et al., 2017), fertilization (Gatti et al., 2018) and harvest (Bramley et al, 2005).

Automation in agriculture is a developing field covering different aspects such as autonomous guidance, including route and field layout planning, crop and environment sensing, and physical interaction with crops (Vougioukas, 2019). Unlike traditional mechanization, robotic solutions form a complement for the human workforce when performing autonomous highly selective operations. As a matter of fact, they may require the use of Artificial Intelligence (AI) and computer vision to detect target regions in the crop environment through application of object detection techniques that are becoming much more popular in agriculture (Kamilaris & Prenafeta-Boldú, 2018; Jha et al., 2019). AI-based systems have recently incorporated artificial neural networks, allowing reliable predictions in response to rigorous training, instead of programming that characterized traditional AI-based methods (Jha et al., 2019). Convolutional Neural Networks (CNNs) are normally used for image classification. Among CNNs, Faster R-CNN (Ren et al., 2017) performs object detection featuring a “Region Proposal Network”, while Mask R-CNN (He et al., 2020) evolved from Faster R-CNN, extending it with an additional feature pyramid network to predict the object mask at the same time as the object bounding box recognition.

Machine Learning (ML) algorithms and frameworks such as the ones mentioned above, are increasingly applied in agriculture. Image segmentation algorithms have been studied (Bargoti & Underwood, 2017) for fruit detection and counting in orchards. Numerous studies on different species have been reported: the number of grapevine berries was assessed by image analysis (Aquino et al., 2018), a computer vision system was developed for driving actions of a kiwifruit robotic harvester (Williams et al., 2020), and a Deep Learning (DL) model based on RGB (Red Green Blue) imagery was fine-tuned to detect and identify different cultivars in mango (Borianne et al., 2019). An on-the-go model aimed at providing an early automated crop-load estimation in vineyards has also been developed (Aquino et al., 2018). Working on Albariño and Barbera cultivars, other authors compared the performances of different deep convolutional neural network architectures and feature spaces by working on images of grapevine clusters (Cecotti et al., 2020).

Grimm et al. (2018) presented a proof of concept for detecting and quantifying plant organs for non destructive yield estimation. This approach is based on automated detection, localization, count and analysis of yield components such as young shoots, inflorescences, and berries. A CNN was created for the semantic segmentation and tested, along with object detection and localization on six different datasets that cover different growth stages of grapevines. Santos et al. (2020) presented a public dataset for grape cluster detection and instance segmentation containing 300 images, bounding boxes, masks and an evaluation of two state-of-the-art methods for object detection, object segmentation and a fruit counting methodology. In the evaluation of the methods the authors considered that Mask R-CNN presented superior results in relation to the YOLO network, but, at the same time they affirmed that the bounding box annotation used to train the YOLO networks is created faster.

Pruning automation is a topic of increasing interest in horticulture. New technologies and developments concerning pruning automation in apple trees have been reviewed (He & Schupp, 2018) and a general framework of an autonomous pruning system led to promising results (You et al., 2020). Recently, Zahid et al. (2021) reviewed the advancements in each core component of a robot for apple tree pruning and provided an exhaustive overview concerning autonomous pruning and harvesting technologies in horticulture. The development of an autonomous pruning system relies upon strong perception systems, motion planning algorithms, robotic arms and specific end-effectors. Computer vision allows plant segmentation, reconstruction and modelling (Tinoco et al., 2021) as well as pruning point detection (Karkee et al., 2014). Scarce information is available for what concerns grapevine architecture over dormancy. Early studies were performed by Mercurio et al. (1989) focusing on a vision-guided block-type robotic grapevine pruner and by McFarlane et al. (1997) working on image analysis algorithms to collect measurements relevant to long-cane winter pruning. A pioneering study developed an image analysis algorithm for spur pruning points identification using an artificial background and black and white images (Gao & Lu, 2006), while Botterill et al. (2017) reconstructed a 3D model of the vine using trinocular stereo cameras in a controlled setting and successively applied an AI algorithm to develop a long-cane pruning scheme. Moreover, a computer vision-based algorithm for grapevine bud detection was presented (Díaz et al., 2018). The approach to the target plant is generally performed using a robotic arm and collisions with non-targeted canes need to be avoided. In this regard, the success of a pruning robot requires reliable and crop-specific motion planning algorithms. In that respect, Magalhães et al. (2019) benchmarked the motion planning algorithms for robotic manipulators in a simulated vineyard. Another essential component of a pruning robot is the end effector, which can use various cutting systems such as pneumatic (Zahid et al., 2020), hydraulic (Vision-Robotics Corporation, 2015) or electric (Botterill et al., 2017). The computer vision system described by Botterill et al. (2017) was then integrated into an over-the-row mobile platform, and the engulfed vine was illuminated with artificial lights and a robotic arm with a collision-free trajectory performed the AI-driven pruning. The major challenges of the prototype were the total execution time, and the limited suitability of the platform to steep slopes and narrow turns. To the best of the authors’ knowledge, a valid and commercially-efficient robotic system for short winter pruning in vineyards is still missing. Moreover, despite the above mentioned advances in sensing, control and manipulation technologies, performances of robotic platforms may be significantly improved in future by coupling technological progress to innovative vineyard management with the final aim to speed up the adoption of robotic solutions towards more efficient, safe and sustainable viticulture (Bloch et al., 2018; Verbiest et al., 2021).

This work aims at fine-tuning and testing (i) a DL-based algorithm for detecting pruning regions (PRs) of spur-pruned grapevines, and (ii) a convolutional neural network allowing plant organ segmentation of dormant grapevines. Moreover, the paper intends analyzing strengths and weaknesses of neural networks depending on different canopy management solutions towards a more effective plant organ segmentation supporting robotized pruning tasks. The study is part of the pipeline for the development of a complete algorithm for cutting-point generation to be implemented on a robotic arm for automated spur-pruning in vineyards. For each plant, the pipeline will use the first network to identify the PRs and then the second network to perform grapevine organ segmentation of the identified PRs.

## Material and methods

To test each step of the proposed pipeline, two experiments were performed: Experiment 1 is about the pruning regions detection with a first Deep Convolutional Neural Network (DCNN), and Experiment 2 is about the plant organ segmentation with a second DCNN.

### Experiment 1, training and testing of the DCNN for PR detection

#### Image collection

**Fig. 1:**
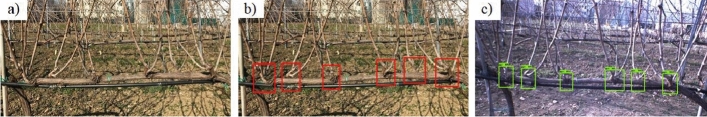
Description of the workflow required for fine-tuning a DCNN for PR identification: Original image (**a**), annotated image by experts for training the neural network by using red bounding boxes (**b**), and example of PR detection through Faster R-CNN with green boxes indicating detected pruning regions(**c**)

During winter, a total of 1215 RGB images were acquired on *Vitis vinifera* L. spur-pruned grapevines from 2 different vineyards characterized by different plant and cordon age (Table S1). In February 2018 and February 2019, 965 and 100 RGB images, respectively, were acquired from *Vitis vinifera* L. cv Merlot grapevines planted in 2014 in an experimental vineyard located in Piacenza (45°02′N, 9°43′E), Italy. Mature vines presented seven 2-node spurs and were planted along a NS-oriented row with 2.1 m × 1.2 m spacing (inter- and intra-row). The cordon was set at 0.9 m above the ground. Images were captured with a resolution size of 1280 × 720 pixels, moving from North to South along the row at a 0.9 m operating distance. In December 2018, 150 RGB images were gathered on eight-year-old *Vitis vinifera* L. cv. Ervi grapevines from a commercial vineyard located at Alseno (44°51′34.70″N, 9°56′E), Italy. Each vine was pruned to six 2-node spurs for a corresponding bud load of 12 nodes/vine. The east-facing vineyard featured EW-oriented rows and a 2.5 m × 0.9 m vine spacing (inter- and intra-row, respectively). Images were acquired West to East with the same depth camera settings as described above. During each acquisition campaign all the images were taken at solar noon under clear sky (Fig. 1a).

#### Data annotation

Pruning target regions of each image were hand-labelled and singularly contained in rectangular bounding boxes by using the COCO Annotator tool (Brooks, 2019). Every annotation included individual spurs avoiding overlapping with adjacent regions, and at least the first 2 basal nodes of each cane (Fig. 1b). The annotated dataset, with a total of 8361 bounding boxes, as part of a fine-tuning process, was subsequently fed to the neural network Faster R-CNN (Ren et al., 2017).

#### Training of the DCNN

The network was fine-tuned from a pre-trained model of Faster R-CNN (Lin et al., 2014), trained with the COCO2017 dataset. The fine-tuning hyperparameters were those related to the neural network structure by default adjusted for the following exceptions: the number of training iterations was changed to 50,000 from the original 270,000, the batch size was changed to 1 from an original value 16, and the decaying learning rate which was set to 0.003 from the start, was changed to 0.0003 at 1000 steps and further decayed to 0.00003 at 2000 steps.

#### Testing of the DCNN

The fine-tuned algorithm was tested in October 2021 on 2 different datasets referred to mature spur-pruned grapevines of diverse cordon age, cultivar and subjected to different growing conditions (Table S1). Accordingly, a batch of 202 frames was acquired in February 2019 on a subset of 5 adjacent Merlot vines (hereafter referred to as Merlot dataset) randomly chosen among those already used for training. The second test dataset, composed of 30 RGB images with a resolution of 4608 × 3456‬ pixels, was obtained in December 2020 in Piacenza with a Nikon Coolpix camera on a set of 15 *Vitis vinifera* L. cv Sangiovese potted grapevines (hereinafter referred to as Sangiovese dataset). The vines were arranged in a single row, trained to a spur-pruned cordon since 2017 with five 2-node spurs and a vine spacing of 0.9 m and a 35° NE-SW orientation. The permanent cordon was located at 0.9 m from the ground. Each plant was entirely photographed once from both sides at cordon height. The acquisition and annotation of both the test datasets considered the same equipment and settings already reported for training (Fig. 1a, b).

**Fig. 2:**
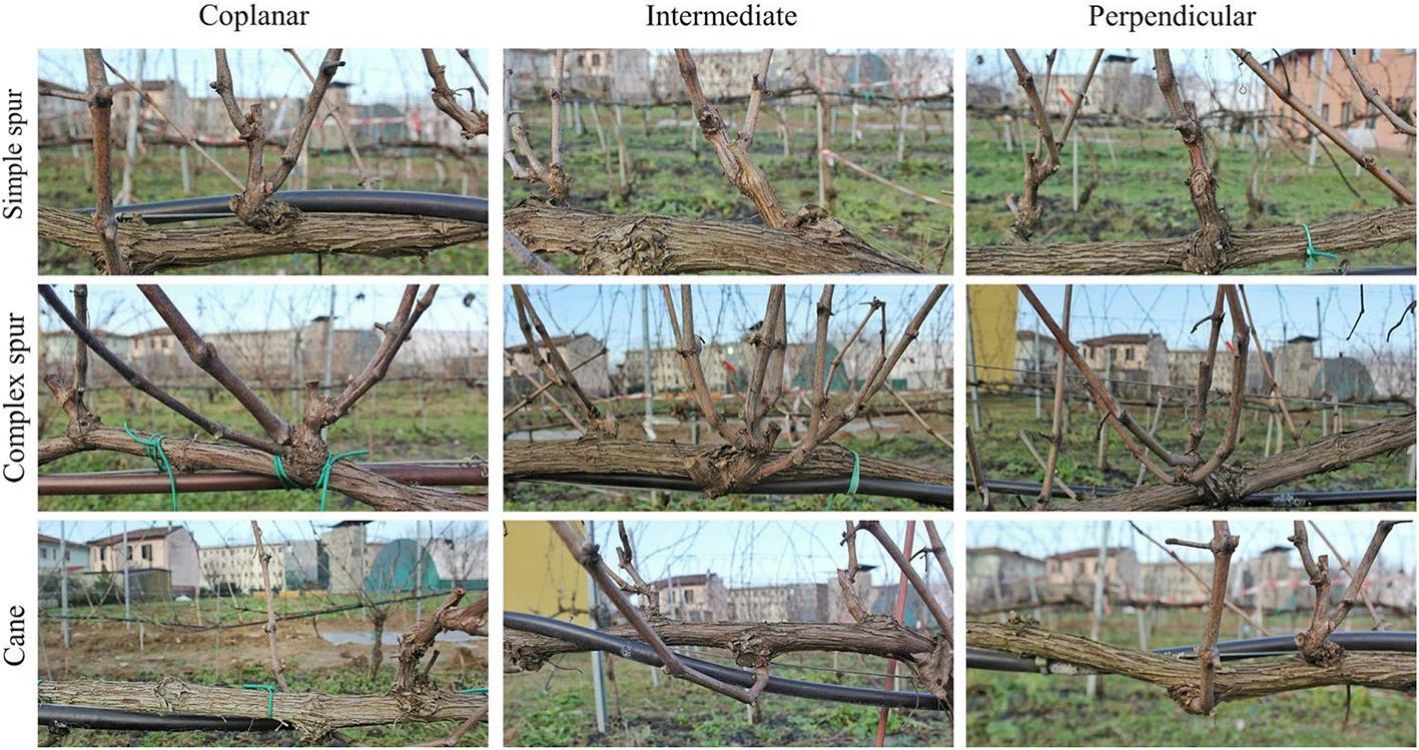
Description of the pruning regions (PRs) of spur-pruned grapevines depending on wood type and orientation. PRs defined as “other” are not reported

For each image, the DCNN predicted the Potential Pruning Regions (PPRs) through bounding boxes and confidence values (Fig. 1c); however, only the detections with confidence > 70% were considered. Additionally, every PR was progressively numbered and described by: wood type (W), visibility (V), and orientation (Or). Wood type included the following categories: cane (cane arising from latent buds on the permanent cordon), simple spur (spur with ≤ 1 shoot/node), complex spur (spur with > 1 shoot/node), and other (PRs not falling in one of previous categories) (Fig. 2). For what concerns visibility, PRs were classified as visible or hidden if occluded by other grapevine organs and/or trellis components. Lastly, orientation provided three categories: coplanar (PR lying on the same plane of the row), perpendicular (PR lying on the vertical plane perpendicular to the row), and intermediate (PR lying on a plane in between coplanar and perpendicular conditions) (Fig. 2).

### Experiment 2, training and testing of the DCNN for grapevine segmentation

#### Image collection

**Fig. 3:**
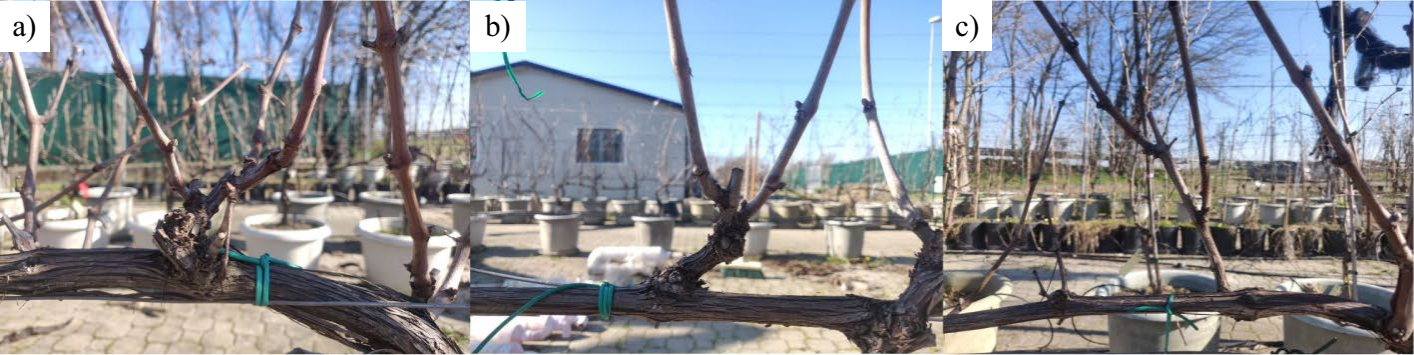
Test set example images from each PR category considered as part of the segmentation network: Control (**a**), Shoot Thinning (**b**) and Light Pruning (**c**)

In March 2021, 148 RGB images were captured with a resolution size of 4608 × 3456 pixels, on the Sangiovese grapevines already considered for experiment 1 (Table S1). To increase the variability among the pruning region complexity, in May 2020 shoot thinning (ST) was performed on 8 out of the fifteen grapevines according to Bernizzoni et al. (2011). The remaining 7 plants acted as an unthinned control (C) (Fig. 3). The acquisition was performed at solar noon, when each PR was individually photographed from a distance of 0.3 m at cordon height; 2 passages per row were performed to consider both the East and West sides. An additional batch of 196 RGB images taken in December 2020 was also considered. Data was randomly captured on the same Sangiovese experimental row with a resolution size of 4608 × 3456 pixels considering different orientation.

#### Data annotation

**Fig. 4:**
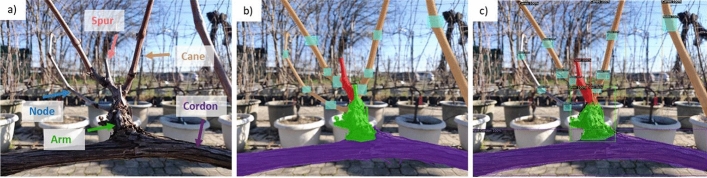
Description of the workflow required for fine-tuning a DCNN for grapevine organ segmentation: Original acquisition with indication of the 5 relevant classes for winter pruning (**a**); annotated image by experts for training the neural network by using 5 different categories (**b**): cordon (purple), arm (green), spur (red), cane (brown), node (blue); and example of PR segmentation through Mask R-CNN (**c**)

The images were annotated using the COCO Annotator tool (Brooks, 2019) and five classes were used to describe the relevant grapevine organs (GO) for pruning purposes: cordon, arm, spur, cane, and node (Fig. 4a). Each grapevine element belonging to the above-mentioned classes was annotated with a polygon, except for nodes that were considered through bounding boxes (Fig. 4b). Polygonal annotation was carried out retracing each element including the outer edge of every organ. To distinguish connected organs within a PR (i.e. arm vs. spur, spur vs. canes) from occlusions and close elements of the background, a few millimeters overlap between annotated areas was kept for contiguous grapevine organs.

#### Training of the DCNN

The network was trained on 119 images using COCO2017 pre-trained model weights for the Mask R-CNN. The default training hyperparameters related to the neural network structure were considered. To adapt the model to the relatively small dataset, the number of training iterations was limited to 50,000, from the original 270,000 and the batch size changed to 2, from the original value of 16.

#### Testing of the DCNN

The original dataset was randomly split into a training dataset (80%) and a test dataset (20%). Accordingly, 29 images of the test dataset were integrated with 31 images collected in December 2020 as part of a preliminary iteration of the neural network (Fernandes et al., 2021). Such a preliminary batch of images considered the highest morphological variability of grapevine pruning regions encompassing unthinned grapevines (C), spurs subjected to early-season shoot thinning (ST), and light pruning (LP) that is generally undesired because favoring acrotony due to the node-count per spur > 2 (Fig. 3). Therefore, in November 2021 the network was tested on a batch of 60 images representing several canopy management conditions hereafter described as treatment (T). The segmentation output (Fig. 4c) was composed of inferences provided with an ID, a class label and the corresponding confidence value, and Intersection over Union (IoU) to quantify the overlap between the annotated organ and the model inference.

#### Evaluation criteria

For each dataset of experiment 1, the network returned bounding boxes identifying the potential pruning regions (PPRs) of the selected images. Consequently, model predictions (PPRs) were compared with actual PRs and three possible outcomes were considered: true positive (TP) when the prediction correctly matched with the corresponding PR; false positive (FP) when the prediction did not correspond to a PR; false negative (FN) in case PRs were not predicted by the DCNN. In addition, FPs were divided into the following 6 categories: arm (old wood growing from the cordon), cane (intermediate portion of a cane), cordon (portion of the permanent cordon of a target vine), next-row trunk (NRT) (intersection between the cordon in the foreground and a trunk in the background), old cuts (OC) (portion of the cordon where previous cuts were performed), post (component of the vineyard trellising). For each FP category the false discovery rate (FDR) was calculated as follows:

1 $${\text{FDR = FP/(TP + FP)}}$$

For each of the 5 classes measured within experiment 2, the output of the grapevine segmentation network was compared to the annotated images. The correctness of a detected grapevine organ was assessed through the IoU overlap with the corresponding ground truth labelling (Girshick et al., 2016; Zhang et al., 2018). The IoU overlap was defined according to the following equation:

2 $${\text{IoU = (A}} \cup {\text{B)/(A}} \cap {\text{B)}}$$

where A stand for hand annotated area and B represents the corresponding inference area.

Within every class, a detected object was assumed as a true positive (TP) when its IoU was higher than 0.5 (Lin et al., 2020). The output was classified as a false negative (FN) when a detected organ did not reach the minimum IoU threshold. The output was classified as false positive (FP) in the case of no overlap with the corresponding ground truth annotation. For FPs, the misclassed grapevine organ or other element was described and considered for further analysis.

In both the experiments, the neural network performances were evaluated through recall, precision and F1 scores, that were calculated for the overall object population of the different datasets according to Kamilaris and Prenafeta-Boldú (2018):

3 $${\text{Recall = TP/(TP + FN)}}$$

4 $${\text{Precision = TP/(TP + FP)}}$$

5 $${\text{F1 score = 2* (TP * FP)/(TP + FP)}}$$

As part of Experiment 1, the same indices were also calculated depending on PR Visibility. In the case of WxV, OrxV, and WxOrxV interactions, mean values of the Recall index were calculated and compared by standard error. In the case of Experiment 2, the performance metrics were calculated based on the grapevine organ (GO), treatment (T), and their interaction (GO × T).

## Results

### Experiment 1

**Fig. 5:**
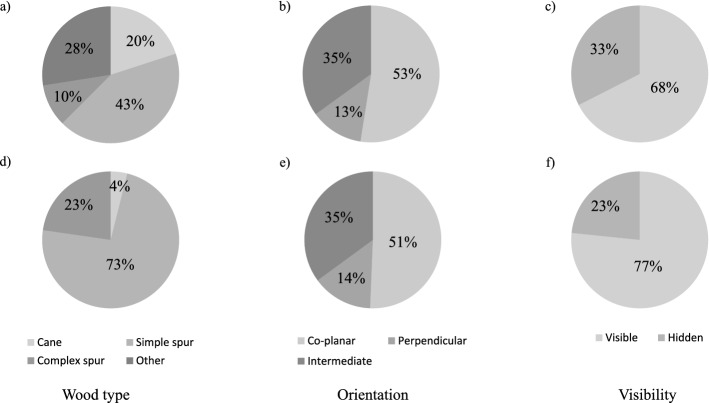
Pruning regions (PRs) breakdown according to Wood Type (**a**, **d**), Orientation (**b**, **e**) and Visibility (**c**, **f**) against the Merlot (**a**–**c**) and Sangiovese (**d**–**f**) datasets. Merlot N = 40, Sangiovese N = 154

The Merlot dataset included 40 pruning regions mostly featured by simple spurs (43%) and coplanar orientation. Simple spurs were also the most common wood type in Sangiovese (73%) where almost half of the PRs were coplanar (51%) with the row’s vertical axis. Moreover, most of the PRs were clearly visible in both the Merlot (68%) and Sangiovese (77%) datasets (Fig. 5).

In Merlot, the PR identification was characterized by lower recall (0.66) and higher precision (0.87) rates while in Sangiovese, the DCNN performances were represented by the following metrics: 0.59 recall, 0.96 precision and 0.73 F1 score (Table 1). Correct PR’s identification was higher in visible spurs with a dramatic recall decrease from 0.72 to 0.53 and from 0.70 to 0.27 when considering occlusions in Merlot and Sangiovese, respectively (Table 2).

**Table 1:** Performance measures of the Faster-RCNN 2.0 vision approach for PR detection against the Merlot and Sangiovese datasets

	Images	#TP	#FN	#FP	Recall	Precision	F1 score
Merlot	202	1007	523	149	0.66	0.87	0.75
Sangiovese	30	100	69	4	0.59	0.96	0.73

*FN* false negative; *FP* false positive; *TP* true positive

**Table 2:** Performance measures of the PR detection model against the Merlot and Sangiovese datasets depending on PR visibility

Dataset	Visibility	#TP	#FN	Recall	Confidence
Merlot	Visible	759	299	0.72	0.82 ± 0.01
	Hidden	248	224	0.53	0.81 ± 0.01
Sangiovese	Visible	89	39	0.70	0.82 ± 0.06
	Hidden	11	30	0.27	0.82 ± 0.04

*FN* false negative; *FP* false positive; *TP* true positive

**Fig. 6:**
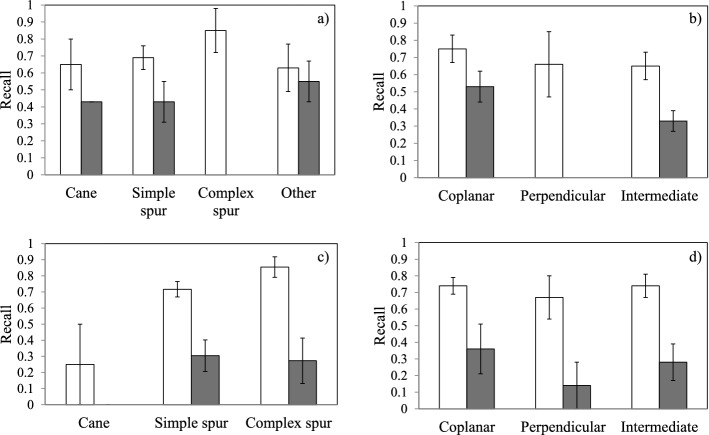
Variation over Wood type (**a**,**c**) and Orientation (**b**,**d**) of the Recall index as a function of PR’s Visibility in the Merlot (top) and Sangiovese (bottom) datasets. Visible and hidden PRs are reported in white and grey, respectively. Bars represent the mean value ± SE

Because of the improvement of DCNN performance fostered by visible PRs, the detection model was then assessed as based on the “wood type x visibility” (WxV) and “orientation x visibility” (OrxV) interactions (Fig. 6). The best detection for Merlot grapevines was reported for visible complex spurs with 0.85 recall followed by visible simple spurs and canes. Simple spurs were associated with the lowest standard error (SE = 0.07) as compared to the other classes (Fig. 6a). Visible coplanar spurs showed the highest detection (0.75 recall) as compared to perpendicular and intermediate PRs (Fig. 6b). Similarly, visible complex spurs of Sangiovese grapevines were associated with the highest recall (0.85) and simple spurs were the second most detected wood type; moreover, consistency of detection performance was proved by relatively low standard errors (0.06 vs. 0.05). Conversely, the same metrics worsened for visible canes showing the lowest recall (0.25) and inconsistent detection (SE = 0.25) (Fig. 6c). Both intermediate and coplanar spurs showed the highest detection (recall = 0.74) as compared to perpendicular PRs (Fig. 6d). Notably, the variability of the recall index calculated for several wood types and orientations for hidden pruning regions was generally higher as compared to visible PRs (Fig. 6).

Some categories such as coplanar complex spurs, intermediate and perpendicular canes, and examples belonging to the category “other” were never observed within the Merlot PRs (Table 3). Visible complex spurs with intermediate orientation were detected with 0.97 recall and a standard error of 0.03. Similarly, recall values were higher than 0.9 for visible perpendicular simple spurs while the detection performance for the same PR with coplanar orientation did not reach 0.75. Irrespective of their orientation, the percentage of TPs associated with hidden simple spurs ranged between 41 and 43% in intermediate and coplanar spurs, respectively (Table 3). Simple and complex spurs were mostly considered in Sangiovese grapevines. When clearly visible, both coplanar and intermediate complex spurs were associated with the highest recall scores (0.85) followed by coplanar simple spurs (0.74). Detection performance for perpendicular and intermediate simple spurs was close to 0.7. Moreover, in both the datasets, the recall index was mostly lower than 50% when PRs were hidden.

**Table 3:** Detection rate of the interactions between Wood Type, Orientation and Visibility in the Merlot (top) and Sangiovese (bottom) datasets

	Wood Type	Orientation	Visibility	
			Visible	Hidden
Merlot	Simple Spur	Coplanar	0.74 ± 0.11	0.43 ± 0.15
		Perpendicular	0.91	–
		Intermediate	0.51 ± 0.06	0.41
	Complex Spur	Coplanar	–	–
		Perpendicular	0.73	0.27
		Intermediate	0.97	0.03
	Cane	Coplanar	0.76 ± 0.12	0.43
		Perpendicular	–	–
		Intermediate	–	–
	Other	Coplanar	–	0.97 ± 0.13
		Perpendicular	–	–
		Intermediate	0.63 ± 0.14	0.29 ± 0.08
Sangiovese	Simple Spur	Coplanar	0.74 ± 0.06	0.38 ± 0.18
		Perpendicular	0.69 ± 0.13	–
		Intermediate	0.67 ± 0.10	0.40 ± 0.16
	Complex Spur	Coplanar	0.85 ± 0.11	0.50 ± 0.50
		Perpendicular	1	1
		Intermediate	0.85 ± 0.09	0.13 ± 0.13
	Cane	Coplanar	0.33	–
		Perpendicular	–	–
		Intermediate	–	–

In Merlot, the false positives were mainly represented by arms and next-row trunks (NRT) with a false discovery rate (FDR) of 6.14% and 4.5, respectively (Table 4). Old cuts (OC), canes and cordons were associated with the following FDR: 1.73, 0.43 and 0.09%, respectively. Only 4 FPs were categorized in Sangiovese grapevines out of the 154 PRs with a negligible impact on the detection performance.

**Table 4:** Description of the FPs detected during the DNN testing

	Class	#FP	Average confidence	FDR (%)
Merlot	Arm	71	0.79 ± 0.16	6.14
	Cane	5	0.77 ± 0.04	0.43
	Cordon	1	0.71	0.09
	NRT	52	0.74 ± 0.23	4.50
	OC	20	0.79 ± 0.04	1.73
Sangiovese	Arm	2	0.74	2.00
	OC	2	0.83 ± 0.04	2.00

*FP* false positive; *FDR* false discovery rate; *NRT* next row trunk; *OC* old cuts

### Experiment 2

The general performances of the segmentation network were described by a recall of 0.81 and a precision of 0.97 with an F1 score of 0.88 (Table 5).

**Table 5:** Overall performance of the neural network for grapevine segmentation with an IoU of 0.5

Count	#TP	#FN	#FP	Recall	Precision	F1 score
1359	1069	258	32	0.81	0.97	0.88

The most recurrent GO in the testing dataset was node followed by cane, spur, arm and cordon (Table 6). False positives related to each class were generally low. The highest recall value was scored by nodes (0.88), followed by cordon and arms (0.81), while spur and cane classes revealed a recall of 0.72 and 0.68, respectively. The precision values ranged from 0.96 (node) to 1 (cordon) with arm and spur segmentations showing intermediate performances.

**Table 6:** Performance measures of the neural network for grapevine segmentation depending on 5 different grapevine organs with an IoU of 0.5

Organ	Count	#TP	#FN	#FP	Recall	Precision	F1 score
Cordon	75	61	14	0	0.81	1.00	0.90
Arm	89	71	17	1	0.81	0.99	0.89
Spur	108	77	30	1	0.72	0.99	0.83
Cane	343	229	107	7	0.68	0.97	0.80
Node	744	631	90	23	0.88	0.96	0.92
Total	1359	1069	258	32			

For canopy management, the most represented category was control (C) followed by shoot thinning (ST) and light pruning (LP) (Table 7). TPs were 493 in C, 487 in ST, and 89 in LP with the highest recall values calculated for grapevine organs subjected to ST (0.85), and relatively lower performances described in C (0.80); moreover the segmentation of the grapevines subjected to light pruning led to the lowest recall. With only 5 wrong inferences, precision was highest in C (0.99), with similar responses described for ST organs despite the higher number of false positives (15). Conversely, although the FPs in LP (12) were relatively similar to ST, the precision was much lower (0.88).

**Table 7:** Performance measures of the neural network for grapevine segmentation depending on canopy management with an IoU of 0.5

Treatment	Count	#TP	#FN	#FP	Recall	Precision	F1 score
C	619	493	121	5	0.80	0.99	0.89
ST	590	487	88	15	0.85	0.97	0.90
LP	150	89	49	12	0.64	0.88	0.74
Total	1359	1069	258	32			

*C* control, *ST* shoot thinning, *LP* light pruning

To investigate if and how vineyard management influences dormant canopy segmentation, the model was tested against each T × GO combination (Table 8). In control vines (C) the cordons were detected with a recall of 0.80. Arm segmentation was described with higher recall (0.87), while the model resulted in poorer performance to identify spurs and canes. No FPs were counted in these grapevine organs, giving a precision of 1. The node class had the highest recall (0.89) having 296 TPs and 37 FNs. The model returned 5 wrong classifications (FPs), lowering precision to 0.98.

**Table 8:** Performance measures of the neural network for grapevine segmentation depending on canopy management and grapevine organs with an IoU of 0.5

Treatment	Organ	Count	#TP	#FN	#FP	Recall	Precision	F1 score
C	Cordon	30	24	6	0	0.80	1.00	0.89
	Arm	39	34	5	0	0.87	1.00	0.93
	Spur	43	31	12	0	0.72	1.00	0.84
	Cane	169	108	61	0	0.64	1.00	0.78
	Node	338	296	37	5	0.89	0.98	0.93
ST	Cordon	34	31	3	0	0.91	1.00	0.95
	Arm	41	34	6	1	0.85	0.97	0.91
	Spur	56	43	13	0	0.77	1.00	0.87
	Cane	141	103	32	6	0.76	0.94	0.84
	Node	318	276	34	8	0.89	0.97	0.93
LP	Cordon	11	6	5	0	0.55	1.00	0.71
	Arm	9	3	6	0	0.33	1.00	0.50
	Spur	9	3	5	1	0.38	0.75	0.50
	Cane	33	18	14	1	0.56	0.95	0.71
	Node	88	59	19	10	0.76	0.86	0.80
Total		1359	1069	258	32			

*C* Control, *ST* shoot thinning, *LP*  light pruning

As expected, shoot thinning (ST) showed a lower count than C for annotated canes and nodes, and a similar number of the annotated elements for cordons, arms, and spurs. When compared to C, in ST grapevines the recall values increased for cordon (0.91), spur (0.77) and cane (0.76) with no or minor changes for nodes (0.89) and arms (0.85), respectively. Although correct inferences in ST proportionally increased as compared to C, the model errors also increased affecting the precision for most of the classes such as arm, cane and node, showing the following values: 0.97, 0.94, and 0.97, respectively (Table 8).

The light pruning (LP) presented a lower number of annotated GO (Table 8). In most cases, FNs were similar to, or higher than TPs. This condition was mirrored by the performance metrics such as recall and F1 score revealing the lowest values within the experiment. Both the recall and F1 score identified poor segmentation performances for arms and spurs (0.33 and 0.38 recall, respectively), and higher sensitivity for node detection (0.76 recall). Precision was mostly affected by count varying between 0.75 (spur) and 1 in the case of cordons and arms where no FPs were detected.

Several elements belonging to the grapevine or to the surrounding environment were associated with wrong predictions such as arms, spurs, canes and nodes (Table 9). Nodes were the most wrongly attributed class since 3.6% of the inferences were FPs. The second most frequent incorrect class attribution concerned canes (3.03%), while wrong segmentation of arm and spurs was limited to 1.39 and 1.28%, respectively.

**Table 9:** Description of the FPs detected during the testing of the neural network for grapevine segmentation with an IoU of 0.5

Detected class	True class	Count	Confidence (Mean)	FDR (%)
Arm	Cane	1	0.91	1.39
Cane	Other object	3	0.89	1.29
Cane	Arm	2	0.95	0.87
Cane	Other grapevine organ	2	0.96	0.87
Node	Other object	11	0.97	1.71
Node	Arm	2	0.99	0.32
Node	Other node	7	0.98	1.10
Node	Other grapevine organ	3	0.99	0.47
Spur	Other grapevine organ	1	0.99	1.28
Total		32		

*FDR* false discovery rate

## Discussion

### Experiment 1, training and testing of the DCNN for PR detection

The fine-tuned network for PR detection of spur-pruned grapevines was tested on 2 datasets representative of different cordon age, cultivar and growing conditions. The overall recall values were relatively similar between the 2 datasets with slightly higher detection rates in Merlot (recall = 0.66) as compared to the younger Sangiovese grapevines (recall = 0.59) (Table 1). Indeed, it must be considered that despite being collected in different years, both training and test datasets for the Merlot included grapevines belonging to the same vineyard, suggesting a higher similarity among the PRs. Conversely, even if referring to whole cordon RGB images, taken from a greater distance from the plant with respect to the training setup, the Sangiovese dataset was totally new as part of the life cycle of the model, proving its consistency. Looking at absolute recall values (Table 1), the system is less powerful than a branch detection model developed in an apple orchard (Zhang et al., 2018) at 70% confidence threshold, where using pseudo-color images, and pseudo-color and depth images led to 0.84 and 0.89 average recall, respectively. Similarly, Sa et al. (2016) described high performances of a sweet pepper detection model based on the combination of RGB and NIR information leading to an F1 score of 0.84. The experiences mentioned in the two citations above, suggest that the PR detection system here presented could be improved by considering a different perception setup, such as the implementation of depth data. Interestingly, the dataset with the lowest recall value was associated with the highest precision (0.96 in Sangiovese) in spite of the negligible detection of wrong elements. Such a condition depends on the relatively high confidence threshold adopted for the study (70%) that limited the TP count and suggests that lowering the confidence would lead to an increased detection rate of the pruning regions. For this reason, a lower confidence rate might be considered for future applications. Moreover, PR’s visibility affected the detection process in both the datasets (Table 2). The significant difference between the detection rates of visible and hidden PRs is due to occlusions, a well-known problem in computer vision and in agricultural applications that are frequently performed in unstructured environments (Yang et al., 2020; Zhang et al., 2020). Getting recall scores higher than 0.7 in visible PRs of both the Merlot and Sangiovese testing datasets is an additional confirmation of the detection model consistency. In addition, the occlusion problem mainly depending on PR-to-PR, cordon-to-PR, and trellis elements-to-PR interactions could be tackled by having both sides of the canopy scanned by the vision system, emerging as relatively easy solution for spur-pruned grapevines where spurs are mainly localized on the upper side of the permanent cordon (Fig. 1a).

When analyzing the DCNN sensitivity as a function of different factors such as wood type, orientation and visibility, recall rates were massively improved for some specific categories, with visible intermediate complex spurs showing the highest values in both the datasets, followed by visible coplanar simple spurs (Table 3). However, complex spurs represented just a minor part of the actual PRs as well as only 13–14% of the annotations in both the datasets had intermediate orientation, while a larger proportion of actual PRs fell in other categories such as simple spurs and coplanar orientation (Fig. 5). In addition, regardless of the dataset, the consistency of the detection performances for visible simple spurs is confirmed by the lower standard error associated with the higher count. The poorer cane detection might be due to their scarce representation in the training dataset that was created by including all the PRs belonging to a given number of grapevines irrespective of their different morphology (Fig. 2). Another interpretation of PR detection results depending on wood type should consider their complexity. In fact, considering individual canes as a major element of a pruning region, the model resulted in better detection of the PRs featured by higher cane numbers suggesting that the DCNN successfully learned how to identify a pruning region based on such a distinctive trait. On the other hand, the same trend would be defined if the model would have just more easily detected bigger pruning regions in terms of encumbrance and area. Similarly, because of the camera orientation considered during the acquisition campaigns, the OrxV interactions resulted in the highest recall values for coplanar PRs, and lower values were obtained for intermediate and perpendicular PRs. In fact, due to their cane orientation, coplanar PRs cover a higher image area compared to intermediate and perpendicular PRs with higher overlapping leading to a higher proportion of occluded pixels (Fig. 2). Merlot WxOrxV interactions revealed detection performances of specific PRs (Table 3). Although they produce the best detection results (recall = 0.97), visible intermediate complex spurs are not discussed here because they are represented by only 2 elements in the dataset. Considering the most frequent categories with a count higher than 4 (Fig. 5), with a recall of 0.74, visible coplanar simple spurs were the best-detected pruning regions. In this regard, DCNN consistency was confirmed by similar performances described for the Sangiovese dataset. Indeed, even though visible coplanar and intermediate complex spurs were associated with the highest recall (0.85), the most represented visible coplanar spurs had the second-highest recall (0.74). In fact, there were 55 visible coplanar simple spurs while only 10 visible perpendicular complex spurs and 13 intermediate complex spurs were considered in the testing dataset. The above mentioned results suggest that the DCNN performance could be improved by either engineering or agronomic adjustments. First, more training data might result in better performance of the deep learning model (Shorten & Khoshgoftaar, 2019); second, improved canopy management in summer can condition the canopy architecture leading to a higher proportion of coplanar simple spurs. As a matter of fact, in 2018 canopy management of the Merlot grapevines was limited to vertical shoot positioning (VSP) and trimming, excluding any selective operation such as shoot thinning. This specific management led to PRs with variable and unpredictable shapes and growth directions increasing the rate of complex spurs and other PRs (Fig. 5). Conversely, shoot selection performed on about 50% of the Sangiovese test vines resulted in a higher proportion of simple spurs increasing, in turn, the frequency of one of the best detected categories (Table 3). Because the VSP system requires two pairs of catch wires placed 40 and 80 cm above the cordon, in both the test datasets only 13–14% of the PRs had a perpendicular orientation, highlighting the role of early shoot positioning and proper catch wire height in promoting coplanar instead of intermediate and perpendicular PRs. In addition, regardless of the best match between detection performance and PR’s morphology, the system showed high reliability in identifying coplanar simple spurs that are supposed to be the best agronomical condition, maximizing canopy efficiency in VSP trained spur pruned grapevines (Smart, 1985; Keller, 2015; Poni et al., 2018).

The main wrong detection was represented by arms (Table 4), the permanent ramifications growing from the cordon whose number and length might increase over years because of wrong pruning strategies. As part of the overall project pipeline, this misclassification could be considered as a correct identification since the PR detection algorithm is expected to be followed by the segmentation network for analyzing the whole region and recognizing 5 different grapevine organs including arms. However, the arm detection was considered as a FP because the annotation acting as true data required the inclusion of the whole PR (Fig. 1). Due to the overlap between the permanent cordon in the foreground with the trunks in the background, NRTs were detected by the model as actual pruning regions representing the second most frequent FP category. The incorrect detection of NRT might be decreased by using depth data to filter the image following the study of Fu et al. (2020), where a 1.2 m threshold was used to separate apple tree canopies from the background to improve apple detection. Considering the project pipeline, a higher precision might be pursued; however, PR detection will be followed by PR segmentation and the exclusion of wrong detections.

### Experiment 2, training and testing of the DCNN for grapevine segmentation.

The current study allowed the fine-tuning and testing of a novel DCNN for grapevine organ identification at 0.5 IoU and 0.7 confidence resulting in the following performance metrics: recall of 0.81 and a precision of 0.97 (Table 5). As already mentioned about PR detection, the current results suggest that assuming a lower confidence would increase the network sensitivity towards the grapevine organs’ identification; as a matter of fact, the general improvement of the detection process would lead to an increased recall at the expense of precision because of the higher number of inferences (TP and FP) regardless of their correctness (Table 5). Recently, Sozzi et al. (2022) used F1 score-confidence threshold and precision–recall curves to identify the best confidence thresholds maximizing automatic bunch detection in white grape varieties using deep learning algorithms. Because the current segmentation network is expected to support grapevine organ identification in a winter pruning perspective, the development of highly performant systems is required to limit the risk of missing pruning regions and cutting points. Indeed, this is well known as spur-pruning over dormancy requires specific cuts (i.e. renewal cuts, cane shortening) to be applied to all the pruning regions along the cordon as well as automated pruning system should exclude any manual follow up of unpruned PRs randomly spread through the vineyard. When considering its sensitivity in detecting the 5 organ classes featuring the grapevine canopy over winter, the DCNN resulted in different performances as reported in Table 6. With a recall of 0.88 (i.e. 88% of the specific annotations identified), nodes were the best detected class showing an important improvement on previous results reported by Dìaz et al. (2018) that processing RGB images through computer vision and machine learning algorithms identified grapevine buds with a maximum recall of 0.45. Because of the grapevine structure, nodes were the most represented class in the test dataset (Table 6). The higher number of nodes in each training image can explain why they are the best-segmented class. Consequently, further improvement of the current DCNN version work will consist in providing more training examples of the under-represented classes such as cordons, arms, and spurs to have a more balanced dataset and consistent results among the 5 classes. In addition to the different abundance of training data the heterogeneous performances describing our segmentation process can be explained by the different GO size (i.e. thickness and width) characterizing a grapevine canopy over dormancy. Indeed, when segmenting indoor images with dense clutter, Badrinarayanan et al. (2017) observed a general lower segmentation accuracy for classes occupying a small part of the image. Data reported in Table 6 describe a higher segmentation rate for bigger organs such as cordons and arms (recall = 0.81) while spurs were less detected (recall = 0.72) because of their thinner structure. The importance in size of target organs is also confirmed when comparing segmentation performances described for arms and spurs; indeed, because a spur might be considered as the natural continuation of an arm, and the ratio between their count approaches 1 in both training and test datasets, the higher detection described for the arms might depend on the more complex structure characterizing a permanent organ older than 2 years as compared to a 2 year old spur (Tassie & Freeman, 1992). Canes, despite being the worst detected organ, by the present algorithm (recall = 0.68) were associated with a higher recall value compared to the results reported by Botterill et al. (2017) with the 2D cane detector (0.49). The generally worse segmentation results obtained for spurs and canes can be linked to the higher probability of getting occlusions. Bigger and isolated organs such as cordons and arms are much less subject to occlusion than spurs, relatively thin and short elements surrounded by canes, and canes which are often crossing each other or self-occluding (Botterill et al., 2017). Precision values in experiment 2 are significantly high because of the low number of false positives for each of the five classes. Results are comparable to segmentation results obtained for other fruit trees. Indeed, when segmenting RGB images of apple trees on trellis wires, Majeed et al. (2020) measured a generally lower F1-score ranging from 0.89 (branch) to 0.95 of the background.

Canopy management greatly affected the segmentation results showing the best detection performances in ST grapevines where only one shoot per node was kept (Table 7). Consequently, a ST canopy has fewer elements to be detected, fewer potential occlusions, and a more standardized canopy that leads to better results when applying computer vision algorithms. However, the three treatments revealed different results in terms of GO segmentation (Table 8). Despite slightly improving the overall performances, C followed the same ranking already described in Table 6 with recall values decreasing in the following order: node > arm > cordon > spur > cane. Segmentation of ST canopies revealed the highest recall values; specifically, cordons (0.91) were followed by nodes (0.89) and arms (0.85). Node segmentation is described by the same recall value. The reason recall does not decrease in C treatment is probably due to a lower frequency of occlusion since nodes could only be masked by very thin organs such as canes. In parallel, nodes were successfully segmented also in LP because their morphology did not differ among treatments, while segmentation performances dramatically decreased for the other organs in response to altered growth patterns and PR’s morphology induced by highly variable spur length. Shoot thinning is a summer pruning technique reducing disease pressure, improving canopy microclimate, vine balance and grape quality to increase sustainability of viticulture (Poni et al., 2018). Moreover, this practice allows more efficient shoot positioning in VSP-trained vines due to the reduced shoot number, making the management of their growth direction and orientation easier and, in turn, facilitating winter pruning operation. ST becomes a quite promising practice in vineyards that will be subjected to automated robotic pruning because of the following reasons: (i) better performances of perception modules such as PR detection and GO segmentation due to the increased proportion of simple spurs and limited frequency of occlusions; (ii) better performances of the manipulation module, by facilitating the motion planning to reach cutting points as well as the end-effector operability; (iii) a significant decrease in cut number per meter of row impacting on robot capacity. On the other hand, such a key role assumed by canopy management supports the idea that, to reach their maximum efficiency, robotic solutions in agriculture need to be coupled with a “robot-ready” orchard (Bloch et al., 2018; Verbiest et al., 2021).

The segmentation network detected few FPs as compared to correct inferences (Table 9). The most recurring error consisted of labeling as a node the Other objects such as a variety of small, round and point-like objects of the image background. The segmentation of “other nodes” as “nodes” mainly included blind buds at the base of longer spurs retained in LP treatment (Fig. 3c). Due to acrotony, distal shoots of an upward spur show preferential growth during the season, inhibiting bud breaking of the lower nodes that lose the possibility to develop new shoots in the next season even if keeping a relatively similar morphology (Keller, 2015). The risk associated with this segmentation is that if the old nodes were counted as real, a pruning algorithm could schedule a wrong cut, targeting a spur instead of a cane.

## Conclusions

In this work, two novel Deep Learning-based models for pruning region detection and canopy segmentation of dormant spur-pruned grapevines were fine-tuned and tested in a real environment. Best detection rates (97%) were obtained for visible intermediate complex spurs, whilst the most frequent visible coplanar simple spurs were detected with 0.74 recall, meaning that the algorithm can get outstanding results, especially on either young vines having a simplified cordon and spur structure, and older vines if subjected to effective canopy management. Conversely, PR’s visibility was the main limiting factor influencing the model, suggesting the occlusion problem might be tackled by scanning PRs from multiple perspectives. Improvements of the proposed network have been discussed as related to training set expansion by including images of spur-pruned grapevines of different age and variety, implementation of depth images, and optimization of the confidence threshold to achieve optimal recall-precision balance for autonomous pruning purposes. Reliable canopy segmentation of dormant spur-pruned grapevines was achieved through a Mask R-CNN network specifically trained for identifying five different grapevine organs: cordons, arms, spurs, canes and nodes. Nodes, arms and cordons were the best detected grapevine organs with more than 80% of correct inferences. The overall network’s performance massively improved when tested on shoot-thinned grapevines, highlighting the important role of canopy management in facilitating the introduction of robotic solutions in agriculture. With the final aim of developing an autonomous, versatile, and commercially viable robot for grapevine winter pruning, future studies will address cutting-point generation and motion planning within each pruning region.

## Supplementary Information

Below is the link to the electronic supplementary material.

Supplementary file1 (DOCX 14 KB)

## Data Availability

The pruning region detection dataset generated and/or analyzed during the presented study is currently not publicly available, but can be requested from the corresponding author on reasonable request. The annotated segmentation dataset is published on the zenodo platform at https://zenodo.org/record/5501784.
