# Supplementary material for: Using deep learning for pruning region detection and plant organ segmentation in dormant spur-pruned grapevines
Source: Precis Agric. 2023 Mar 22:1–23. Online ahead of print. doi: 10.1007/s11119-023-10006-y (PMC10032262; doi:10.1007/s11119-023-10006-y)
Supplement: Supplementary file 1 — Supplementary file1 (DOCX 14 KB) [file 11119_2023_10006_MOESM1_ESM.docx]

S1 – Description of the plant material characterizing the datasets used for training and testing two different Deep Convolutional Neural Networks for pruning region detection and plant organ segmentation in dormant spur-pruned grapevines.

| Variety | Site | Planting year | Growing conditions | Spurs per vine | Cordon age - training dataset (years) | Cordon age - testing dataset (years) | Experiment |
| --- | --- | --- | --- | --- | --- | --- | --- |
| Merlot | Piacenza | 2014 | Field-grown | 7 | 1 | 2 | 1 |
| Ervi | Alseno | 2011 | Field-grown | 6 | 2 | - | 1 |
| Sangiovese | Piacenza | 2014 | Potted-vines | 5 | 4 | 4 | 1,2 |
